# Cytomegalovirus (CMV) Central Nervous System Infection and Recurrent Posterior Circulation Ischemic Strokes in an Immunocompetent Patient

**DOI:** 10.7759/cureus.115961

**Published:** 2026-09-08

**Authors:** Sara Azhar, Hajar Naciri Darai, Dahab Ouhabi, Houyam Tibar, Wafa Regragui

**Affiliations:** 1 Department of Neurology B and Neurogenetics, Specialities Hospital, Ibn Sina Teaching Hospital, Mohammed V University in Rabat, Rabat, MAR

**Keywords:** cytomegalovirus infection, headache, immunocompetent individuals, pcr, stroke

## Abstract

Cytomegalovirus (CMV) infection in an immunocompetent adult is rarely symptomatic. Neurological manifestations are uncommon and are predominantly characterized by meningitis, myelitis, and acute polyradiculoneuritis. We report a rare case of an immunocompetent patient who presented with prolonged headaches complicated by recurrent posterior circulation ischemic strokes associated with intracranial stenoses of the basilar trunk and posterior cerebral arteries. Analysis of his cerebrospinal fluid (CSF) revealed lymphocytic meningitis with moderate elevation of the CSF protein level, normal CSF glucose level, and detection of the CMV genome by polymerase chain reaction (PCR). Viral load quantification showed high levels in both blood and CSF; serology was consistent with probable primary CMV infection. A comprehensive assessment ruled out any other causes of stroke. Treatment with ganciclovir was initiated, which led to normalization of the CSF; however, the clinical course was marked by the onset of meningeal hemorrhagic complications with triventricular hydrocephalus, followed by gastrointestinal and urinary hemorrhagic complications, which caused the death of the patient. Our case highlights the possibility of severe CMV central nervous system infection in immunocompetent individuals and the importance of considering testing for CMV in cases of persistent unexplained headaches accompanied by recurrent ischemic strokes, inflammatory CSF, or intracranial vasculopathy to avoid diagnostic and therapeutic delays, which can be fatal.

## Introduction

Cytomegalovirus (CMV) infection is a widespread viral infection caused by human herpesvirus type 5 (HHV-5), which belongs to the Herpesviridae family. Primary infection occurs through transmission of the virus via breast milk, mucous membranes, or blood contact. The virus establishes a persistent, lifelong latent state with the potential for reactivation, particularly in immunocompromised individuals [1,2]. In immunocompetent individuals, the infection is most often asymptomatic or presents as a mild mononucleosis-like syndrome. In contrast, in immunocompromised patients, particularly those who have undergone solid organ transplantation, hematopoietic stem cell transplant recipients, and patients infected with human immunodeficiency virus (HIV), CMV can lead to severe visceral involvement. The organs most frequently affected are, first and foremost, the retina (retinitis), the gastrointestinal tract (esophagitis, gastritis, or colitis), followed by the lungs (interstitial lung disease), the liver (hepatitis), and the nervous system [2,3].

Neurological complications are predominantly characterized by subacute presentations of encephalitis, ventriculoencephalitis, transverse myelitis, or polyradiculopathy [1,4]. They are also rarely reported in immunocompetent individuals, particularly in the context of multiorgan involvement [3], in which gastrointestinal and urinary hemorrhages may occur due to tissue ulceration and, more rarely, intracranial hemorrhages due to inflammatory microvascular damage [5-7]. CMV-associated vasculitis is also a rare manifestation of CMV infection, resulting from direct infection of vascular endothelial cells or an immune-mediated response, leading to vascular inflammation and ischemia [8].

Diagnosis is primarily based on the detection of CMV DNA in the cerebrospinal fluid (CSF) by polymerase chain reaction (PCR); a positive result still requires interpretation within the clinical syndrome and differential diagnosis. Treatment consists of intravenous ganciclovir or foscarnet, or their combination, and should be initiated promptly once CMV infection is suspected [1].

We report a rare case of severe CMV central nervous system (CNS) infection in an immunocompetent patient. This case sheds light on the importance of considering CMV testing in cases of unexplained chronic headaches accompanied by recurrent ischemic strokes, especially with inflammatory CSF or intracranial vasculopathy, in immunocompetent individuals. The prognosis depends on early diagnosis and initiation of treatment.

## Case presentation

This is a 63-year-old patient with no prior malignancy, transplant, corticosteroid or immunosuppressive exposure, diabetes, or renal or hepatic disease; his full blood count (with differential and absolute lymphocytes) was normal. He had no cardiovascular risk factors (no smoking, hypertension, dyslipidemia, or prior vascular disease). He had been complaining for four months of fluctuating, diffuse headaches without radiation that had been progressively worsening, with no triggering factors, and that responded partially to analgesics (Table 1). There were no associated symptoms, in particular no vomiting, visual disturbances, jaw claudication, or deterioration in general health. His neurological examination was normal. One month later, the patient presented with left-sided hemiplegia and dysarthria due to a right paramedian pontine stroke with intracranial stenosis of the basilar artery (Table 1). Cardiac investigations (electrocardiogram, transthoracic and transesophageal echocardiography, CT angiography of the supra-aortic trunks) were normal. Inflammatory and immunological tests (including C-reactive protein, erythrocyte sedimentation rate, antineutrophil cytoplasmic antibodies, soluble antigen and antiphospholipid testing, anti-Sjögren’s syndrome-related antigen A and antigen B antibodies, serum angiotensin-converting enzyme, calcium and phosphate levels, and 24-hour urinary protein excretion) were normal. The patient was placed on dual antiplatelet therapy following a diagnosis of intracranial stenosis. However, the headaches worsened over time, becoming so severe as to cause insomnia and proving resistant to analgesic treatment. He was admitted to our department for management.

**Table 1:** Timeline of clinical events

Pathological event	Diffuse persistent headaches	Right paramedian pontine stroke	Left lateral pontine stroke	Multiorgan hemorrhagic complications
Month/year	November 2023	December 2023	January 2024	January 2024

His clinical condition worsened 20 days later with the sudden onset of vertigo, revealing a left lateral pontine stroke with stenosis extending from the basilar artery to the posterior cerebral arteries (Figure 1; Table 1).

**Figure 1:**
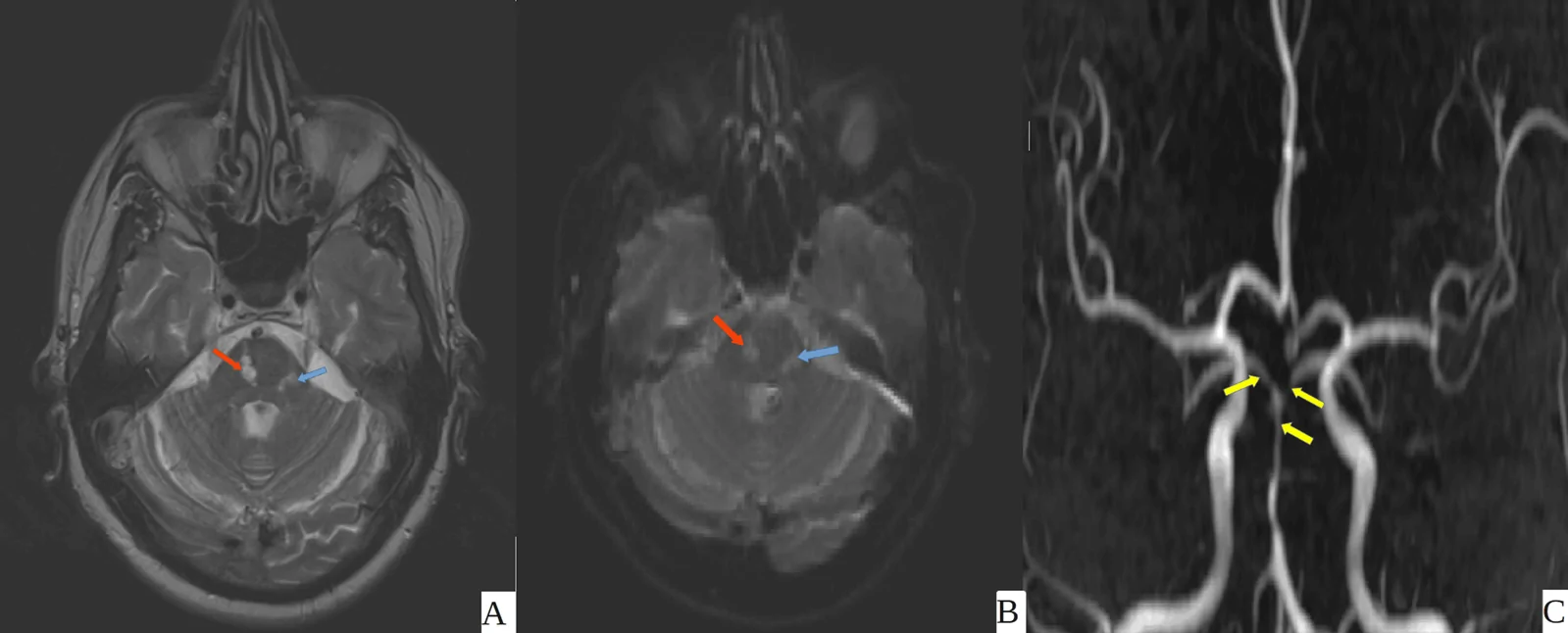
Brain MRI sequences (A: T2-weighted imaging, B: diffusion-weighted imaging, C: TOF) Left lateral acute pontine stroke (blue arrows) and older right paramedian stroke (red arrows). Stenosis of the basilar trunk and posterior cerebral arteries (yellow arrows). TOF, time-of-flight

Analysis of the CSF revealed pleocytosis of 100 cells/mm³ consisting of lymphocytes, an elevated CSF protein level of 1 g/L, and a normal CSF glucose level of 0.8 g/L, as well as detection of the CMV genome on the PCR panel for infectious cerebral encephalitis (including Escherichia coli K1, Haemophilus influenzae, Listeria monocytogenes, Neisseria meningitidis, group B Streptococcus, Streptococcus pneumoniae, herpes simplex virus type 1, herpes simplex virus type 2, varicella-zoster virus, human herpesvirus 6, enterovirus, human parechovirus, and Cryptococcus neoformans/gattii). CMV viral load was high in both blood and CSF; serology was consistent with a suspected primary CMV infection (IgM+, IgG-). Serology tests for hepatitis B virus (HBV), hepatitis C virus (HCV), syphilis (Treponema pallidum hemagglutination assay (TPHA)/Venereal Disease Research Laboratory (VDRL)), and HIV were negative. Liver and renal function tests were normal. The chest X-ray showed no signs of pneumonia or ground-glass opacities.

Treatment with intravenous ganciclovir was initiated at a dose of 5 mg/kg every 12 hours. Antiviral treatment was monitored by complete blood counts and renal function. The coagulation profile was normal, and antithrombotic therapy was stopped. The course of the illness was marked by normalization of the cytological and biochemical CSF analysis (pleocytosis: 1 cell/mm³, protein level: 0.35 g/L, glucose level: 0.8 g/L); however, it was complicated five days later by multiorgan hemorrhagic complications: subarachnoid hemorrhage with triventricular hydrocephalus (Figure 2; Table 1), requiring an emergency external ventricular shunt, followed by massive urinary and gastrointestinal hemorrhage, which led to the patient’s death by hypovolemic shock.

**Figure 2:**
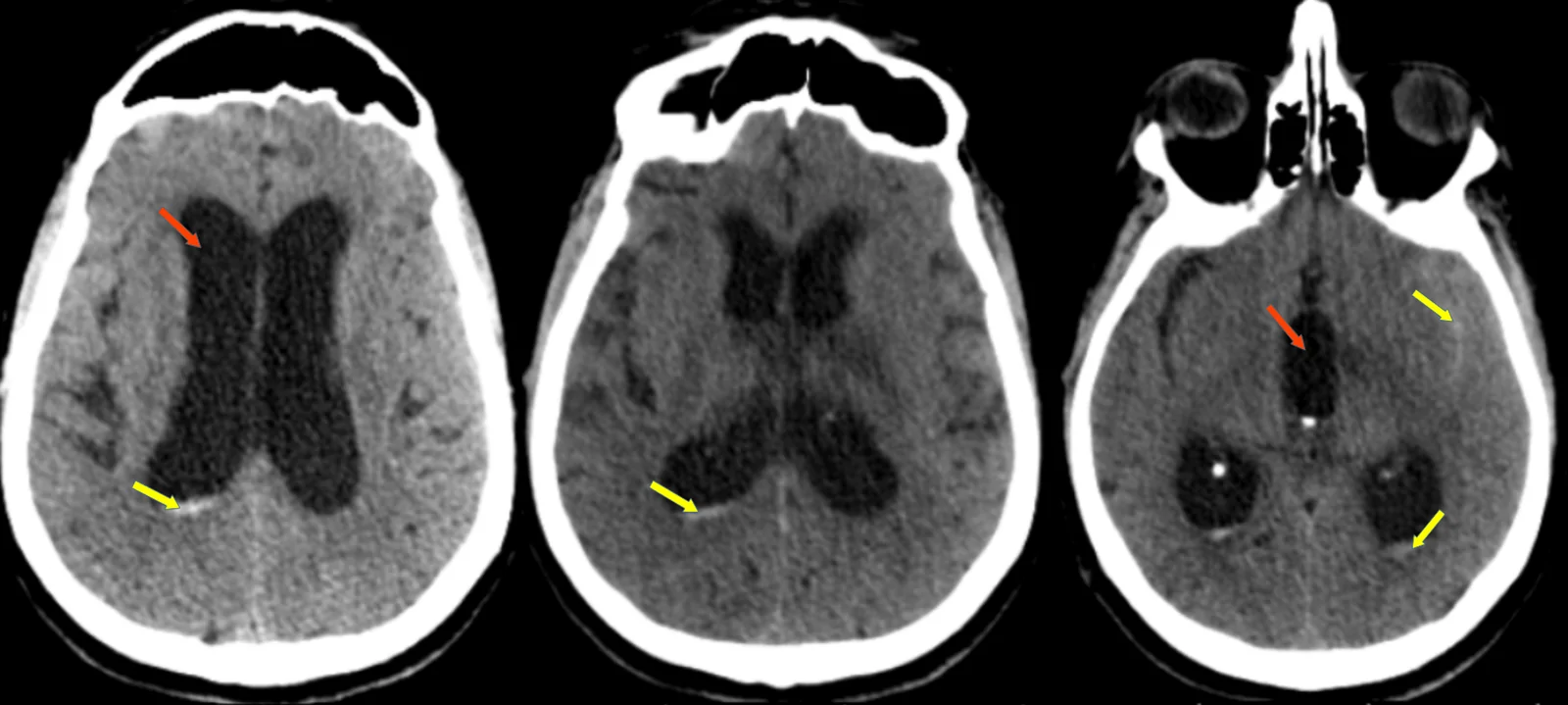
Brain CT scan Triventricular hydrocephalus (red arrows); subarachnoid hemorrhage with intraventricular hemorrhage (yellow arrows).

## Discussion

Our patient presented with headaches and recurrent posterior circulation ischemic strokes associated with intracranial arterial stenosis, which led to the diagnosis of CMV CNS infection complicated by multiorgan involvement. The diagnosis was mainly confirmed by the detection of CMV DNA in the CSF via PCR [1]. The CSF study showed moderate lymphocytic pleocytosis and an elevated CSF protein level with a normal CSF glucose level, which was consistent with the viral etiology of the infection and did not provide evidence of other common infectious causes of CNS infection. Brain MRI, including time-of-flight (TOF) angiography, did not support a diagnosis of reversible cerebral vasoconstriction syndrome and showed no evidence of intracranial atherosclerosis or arterial dissection. Primary angiitis of the CNS was considered unlikely based on the clinical and imaging findings, although it could not be definitively excluded because a brain and meningeal biopsy was not performed.

CMV CNS infection was revealed by headaches that were initially intermittent and moderate, with no meningeal syndrome, focal signs, or systemic signs; this did not prompt extensive investigation and led to a delay in diagnosis of more than four months. Indeed, headaches can be a manifestation of the primary infection in immunocompetent individuals or in cases of CNS involvement. Although their exact prevalence has not been accurately quantified in epidemiological studies, headaches are regularly reported among the nonspecific symptoms of mononucleosis-like viral syndrome and constitute a common presenting symptom in documented neurological forms [3].

From a pathophysiological perspective, headaches are thought to result from several complementary mechanisms. On the one hand, the neurotropism of CMV, which may involve infection of cerebral endothelial cells, astrocytes, and glial cells, could trigger a local inflammatory response characterized by the production of pro-inflammatory cytokines, including interleukin-6 (IL-6) and tumor necrosis factor-alpha (TNF-α). These inflammatory mediators may contribute to the activation of intracranial nociceptive pathways. This neuroinflammatory response may also be associated with meningitis or ventriculoencephalitis, potentially resulting in meningeal irritation, a mechanism classically implicated in the pathogenesis of headache [9].

In our patient, the clinical course was characterized by the occurrence of two ischemic strokes associated with intracranial arterial stenosis. Indeed, ischemic stroke has been reported in the context of CMV infection, although the underlying mechanisms remain incompletely understood. One proposed mechanism involves CMV-induced endothelial activation and inflammation, with increased expression of pro-inflammatory mediators such as IL-6 and TNF-α, which may promote leukocyte recruitment and contribute to vascular inflammation. CMV infection may also affect vascular smooth muscle cells, potentially promoting their proliferation and migration and thereby contributing to intimal thickening and atherosclerotic lesion progression. In addition, CMV has been proposed to promote a prothrombotic vascular environment through endothelial dysfunction and enhanced coagulation activation, which could facilitate thrombus formation, particularly in the presence of preexisting vascular lesions [10-12].

Chronic headaches may, in some cases, precede the onset of a stroke and reflect an underlying progressive cerebrovascular condition. In particular, they are described as "sentinel headaches" associated with arterial dissection, progressive stenosis, or inflammatory or infectious vasculopathy. In such situations, the headache may indicate vascular instability or a prothrombotic state, occurring a few days to several weeks before the ischemic event [3,13], which could be consistent with our patient’s clinical presentation.

Severe primary CMV infection can rarely occur in immunocompetent individuals despite an apparently intact immune system. In these cases, severe disease may result from extensive viral replication and dissemination before effective CMV-specific immunity is fully established, combined with an excessive or dysregulated immune response. Viral immune-evasion mechanisms and host or viral genetic factors may also influence the severity of primary infection [14].

Our patient underwent a comprehensive cardiovascular and immunological work-up following the onset of his first ischemic stroke, which was negative. He was placed on dual antiplatelet therapy, as the ischemic stroke had been attributed to intracranial arterial stenosis. A recurrence of the ischemic stroke, again in the pons, while he was on preventive treatment, prompted a CSF analysis, which revealed CMV meningitis.

Following the initiation of antiviral treatment with ganciclovir, the CSF profile returned to normal, but the patient developed multiorgan complications, which were considered a consequence of the virus itself or the inflammatory reaction after viral infection. The optimal antiviral treatment strategy for CMV CNS infection remains incompletely defined, as clinical data on antiviral efficacy are limited. Current guidelines recommend an initial three-week course of intravenous ganciclovir combined with intravenous foscarnet, as ganciclovir monotherapy appears to have limited efficacy in these cases. This is followed by ganciclovir monotherapy for an additional three weeks in immunocompetent patients and six weeks in immunocompromised patients. During ganciclovir therapy, complete blood counts and renal function should be closely monitored because of its hematological and renal toxicity. Treatment response should be assessed primarily on clinical grounds, with antiviral therapy continued until clinical improvement and clearance of CMV DNA from the CSF. However, the optimal timing for repeat CSF PCR testing has not been standardized. In the case of our patient, he received ganciclovir alone due to the unavailability of foscarnet. The progression of visceral involvement may be linked to the delayed effectiveness of antiviral drugs, high viral load, or the emergence of resistant strains. Cidofovir is a second-line alternative in cases of resistance or intolerance to first-line treatments [3,15,16].

In CMV infection, mortality is observed mainly in immunocompromised patients. The main factors associated with an increased risk of death are advanced age, severe lymphopenia, initial clinical severity, CMV pneumonitis, and opportunistic coinfections. Multiorgan involvement (pulmonary, neurological, gastrointestinal, hepatic, and retinal) is also an unfavorable prognostic factor [3,17]. Our patient presented with multiorgan hemorrhagic complications, including massive urinary and gastrointestinal hemorrhage, which led to his death from hypovolemic shock.

## Conclusions

Neurological presentations of CMV infection in immunocompetent individuals are rare and often associated with delayed diagnosis and treatment, which can be fatal in some severe cases.

This infection may present as chronic headaches; therefore, routine screening for CMV should not be limited to immunocompromised patients and should be considered in cases accompanied by recurrent unexplained ischemic strokes, especially with inflammatory CSF or intracranial vasculopathy. Its prognosis depends partially on the early initiation of treatment with ganciclovir and/or foscarnet; the combination of these two drugs is recommended in severe cases.
